# Supplementary material for: F3 Expression Drives Sensitivity to the Antibody-Drug Conjugate Tisotumab Vedotin in Glioblastoma
Source: Cancers (Basel). 2025 Feb 27;17(5):834. doi: 10.3390/cancers17050834 (PMC11898980; doi:10.3390/cancers17050834)
Supplement: Supplementary file 1 [file cancers-17-00834-s001.zip › cancers-3460217-supplementary.pdf]

Supplementary Figure S1

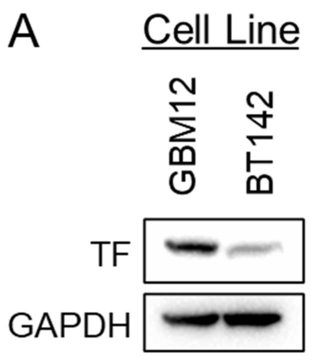

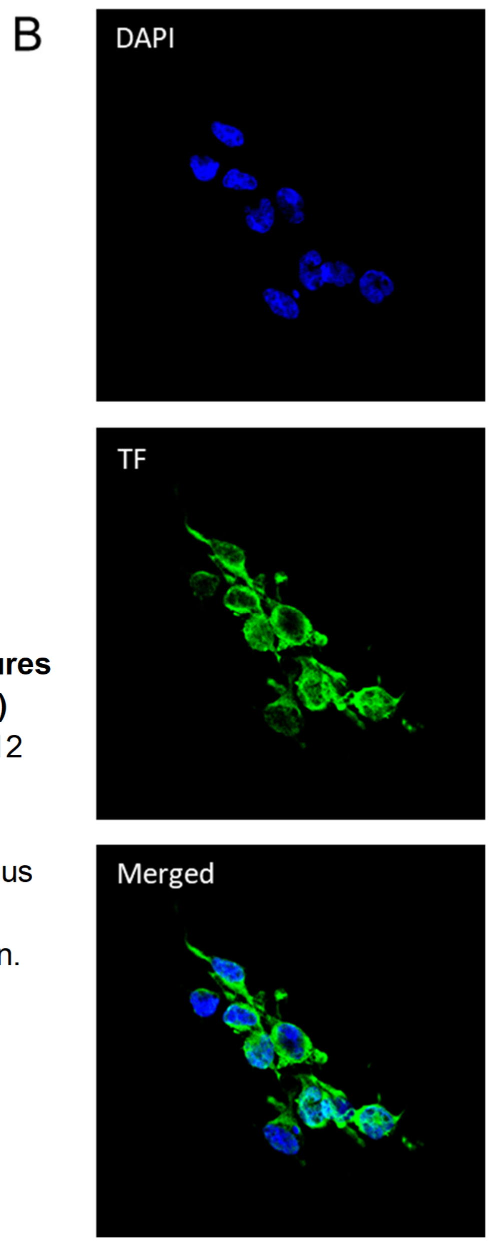

Tissue Factor is highly expressed in GBM12 cultures but exhibits low expression in BT142 cultures. (A) Immunoblot on whole cell lysates derived from GBM12 and BT142 cell cultures showing Tissue Factor and GAPDH expression. **(B)** Immunocytochemistry of GBM12 cell cultures showing cytoplasmic/membranous staining of Tissue Factor counterstained to a DAPI nuclear stain. Images were taken at 40x magnification.
